# On the Robustness of Multiscale Indices for Long-Term Monitoring in Cardiac Signals

**DOI:** 10.3390/e21060594

**Published:** 2019-06-15

**Authors:** Mohammed El-Yaagoubi, Rebeca Goya-Esteban, Younes Jabrane, Sergio Muñoz-Romero, Arcadi García-Alberola, José Luis Rojo-Álvarez

**Affiliations:** 1 Department of Signal Theory and Communications, Telematics and Computing Systems, Rey Juan Carlos University, 28933 Fuenlabrada, Spain; 2 GECOS Lab, ENSA, Cadi Ayyad University, 40000 Marrakech, Morocco; 3 Center for Computational Simulation, Universidad Politécnica de Madrid, 28040 Pozuelo, Spain; 4 Hospital Clínico Universitario Virgen de la Arrixaca de Murcia, 30120 Murcia, Spain

**Keywords:** nonlinear dynamics, multiscale indices, cardiac risk stratification, Holter, long term monitoring, multiscale entropy, multifractal spectrum, multiscale time irreversibility

## Abstract

The identification of patients with increased risk of Sudden Cardiac Death (SCD) has been widely studied during recent decades, and several quantitative measurements have been proposed from the analysis of the electrocardiogram (ECG) stored in 1-day Holter recordings. Indices based on nonlinear dynamics of Heart Rate Variability (HRV) have shown to convey predictive information in terms of factors related with the cardiac regulation by the autonomous nervous system, and among them, multiscale methods aim to provide more complete descriptions than single-scale based measures. However, there is limited knowledge on the suitability of nonlinear measurements to characterize the cardiac dynamics in current long-term monitoring scenarios of several days. Here, we scrutinized the long-term robustness properties of three nonlinear methods for HRV characterization, namely, the Multiscale Entropy (MSE), the Multiscale Time Irreversibility (MTI), and the Multifractal Spectrum (MFS). These indices were selected because all of them have been theoretically designed to take into account the multiple time scales inherent in healthy and pathological cardiac dynamics, and they have been analyzed so far when monitoring up to 24 h of ECG signals, corresponding to about 20 time scales. We analyzed them in 7-day Holter recordings from two data sets, namely, patients with Atrial Fibrillation and with Congestive Heart Failure, by reaching up to 100 time scales. In addition, a new comparison procedure is proposed to statistically compare the poblational multiscale representations in different patient or processing conditions, in terms of the non-parametric estimation of confidence intervals for the averaged median differences. Our results show that variance reduction is actually obtained in the multiscale estimators. The MSE (MTI) exhibited the lowest (largest) bias and variance at large scales, whereas all the methods exhibited a consistent description of the large-scale processes in terms of multiscale index robustness. In all the methods, the used algorithms could turn to give some inconsistency in the multiscale profile, which was checked not to be due to the presence of artifacts, but rather with unclear origin. The reduction in standard error for several-day recordings compared to one-day recordings was more evident in MSE, whereas bias was more patently present in MFS. Our results pave the way of these techniques towards their use, with improved algorithmic implementations and nonparametric statistical tests, in long-term cardiac Holter monitoring scenarios.

## 1. Introduction

Sudden Cardiac Death (SCD) has been defined as the unexpected natural and cardiac originated death within a short period of time, given at less than 1 h from the onset of symptoms if witnessed or within 24 h of having been observed alive if unwitnessed, in a person with no prior condition that could be considered as fatal [1,2,3]. There are other causes of sudden death, but cardiac is the most usual origin, specifically including: (a) Arrhythmic causes, such as ventricular tachycardia (VT), ventricular fibrillation (VF), or asystole; (b) several other structural heart disease origins, for instance, those ones corresponding to congenital heart disease; and (c) abnormal function of the autonomous nervous system (ANS), which is not itself a death cause, but it can promote others such as arrhythmic or hypertensive death [4]. The SCD mechanism in the last case is often VT or VF. Given the incidence of SCD as major cause of mortality in the world, methods have been proposed aiming to provide risk stratification tools for cardiac patients [5]. SCD episodes can happen not only in patients with coronary or cardiomyopathic disease, but also they can occur in people with no previous heart alteration, which makes the risk stratification extremely complex. The prognostic significance of noninvasive studies and the efficacy of the therapeutic actions have been pointed to be etiology dependent [6]. The most widely used SCD-risk marker in the clinical practice is the Left Ventricular Ejection Fraction (LVEF), but given its low specificity, many other techniques have been proposed. A relevant subset of them is given by the computational indices that are obtained from the signal analysis of the Electrocardiogram (ECG), including a variety of proposed biomarkers such as late potentials, heart rate variability (HRV), T-wave alternans, or deceleration capacity. The interested reader can refer to [7] for a detailed review on issues related with signal processing, technology transfer, and scientific evidence for all of them.

Many of these SCD markers are obtained in a Holter recording, which is a diagnostic tool consisting of 24 to 48 h signal registers in two or three chest leads to be subsequently processed by using a computer program, so that a variety of cardiac events can be identified by the clinician. Probably one of the most scrutinized markers of SCD risk from Holter recordings is HRV, which measures the time changes between consecutive cardiac beats [8]. Its interest partially comes from its non-invasive nature and its easy for analysis, only needing to know the time instants of the beat occurrences. The heart does not behave like a periodic oscillator, but instead its rhythm is modulated by the ANS, and the simultaneous actuation of its two branches (sympathetic and parasympathetic) causes dynamic oscillations of the cardiac frequency, producing the presence of HRV [9]. Among the many methods that have been proposed in the literature to quantify the HRV indices, the nonlinear methods extract relevant information from HRV signals in terms of their complexity. Nonlinear indices are based on the underlying idea that fluctuations in the between-beat intervals (also known as RR intervals) can exhibit characteristics that are well known from Complex Dynamic Systems Theory, and broadly speaking, healthy states are expected to correspond to more complex patterns than pathological states. However, some pathologies are associated with highly erratic fluctuations with statistical properties resembling uncorrelated noise [7], and traditional algorithms could yield higher irregularity indices for such pathological signals when compared to healthy dynamics, even though the latter represent more physiologically complex states [10]. This possible inconsistency may be due to the fact that traditional algorithms are based on single scale analysis, and they can not take into account the complex temporal fluctuations inherent to healthy physiologic control systems. It is usual that studies based on 1-day Holter monitoring [11] envision that relevant information could be obtained from longer duration recordings, however, few studies [12,13] have scrutinized nonlinear indices in several-day Holter monitoring, despite its current and increasing availability in the clinical practice. Note in the following that, whereas some authors refer to long-term Holter as those with duration about 24 h, we will use long-term to refer to the Holter recordings when measured for several days throughout this paper.

Several of the nonlinear HRV measurements (based either on Chaos Theory, Information Theory, or Fractal Theory) have been paid special attention according to the electrophysiological hypothesis that the long-term regulation is a homeostatic yet dynamical equilibrium, which can be expected to be complex and multi-cause enough to require a set of indices that should be calculated at different scales. This has motivated the extension of several of those indices to what can be called their *multiscale* versions. Remarkable examples of this effort are the Multiscale Entropy (MSE) method [14,15,16,17], the Multiscale Time Irreversibility (MTI) method [15], and the Multifractal Spectrum (MFS) method [18,19]. MSE has been applied to predict stroke-in-evolution in acute ischemic stroke patients using one-hour ECG signals during 24 h [20], and also recently to predict vagus-nerve stimulation outcome in patients with drug-resistance epilepsy, who were found to have lower preoperative HRV than controls [21]. Different preprocessing stages have been proposed for it, including the use of MSE in the first difference of RR-interval time series instead of the series itself, yielding better statistical support and discrimination capabilities between Congestive Heart Failure (CHF) and control groups [22]. Some authors consider the MSE algorithm biased for two reasons: First, the similarity criteria is fixed for all scales, whereas coarse grained time series variance has been pointed to decrease with the scale; And second, spurious oscillations are introduced due to the suboptimal procedure for eliminating the fast temporal scales of the time series. Accordingly, a modified algorithm was proposed [23], so-called the refined MSE (RMSE), in order to overcome these limitations, and it was tested in simulations and in 24-h HRV signals from aortic stenosis and control groups. The use of RMSE did not allow to make inferences that could not be made by MSE with real data, however, simulations showed that RMSE can be a more reliable method for the assessment of entropy-based irregularity. A comparative study between MSE and RMSE was performed in [24] confirming that despite the differences they both present similar tendencies with scale factor. MTI has been applied to HRV and blood preasure variability (BPV) signals concluding that TI of beat-to-beat HRV and BPV is significantly altered during orthostasis [25]. In addition, recent interest has been raised in the use of some of the multiscale indices in the analysis of atrial fibrillation (AF) dynamics, which has been scrutinized in the context of ischemic stroke prediction in patients with permanent AF [26].

We can say that cardiac long-term monitoring (LTM) has been technologically achieved, and previous studies exist which have scrutinized the value of these recordings in simple and well-known clinical indices from practice, such as the number or rate of ectopic beats or the number or rate of different cardiac events [27]. However, algorithm robustness should be paid attention if deeper physiological and pathological information is to be extracted from nonlinear multiscale indices, in order to be sure about their reliability when working with long series in populational data, and to our best knowledge, few previous works can be found noting this point with attention. Therefore, we propose here to study the robustness of nonlinear multiscale HRV measurements to characterize different cardiac health states in LTM recordings.

Specifically, we made a comparative analysis of the three mentioned multiscale methods on a database consisting of patients with 7-day Holter in two cardiac conditions, namely, CHF and AF [28]. This study aims to give basic knowledge on the usefulness and current limitations of these methods towards their future and principled use for SCD risk stratification. For this purpose, a nonparametric statistical test is proposed in order to compare and give cut-off comparison levels between two different situations in terms of the confidence intervals (CI) for the median difference across multiscale representations of poblational representations. This method can be used either for establishing comparisons among patients or subjects with different conditions, or to scrutinize the impact of preprocessing or data length on the statistical properties of the multiscale representations. The procedure can be seen as an extension of previously used statistical comparisons [20,21] in terms of nonparametric bootstrap tests for confidence bands [12,29].

The structure of the paper is as follows. In the next section, the fundamentals of the multiscale methods selected here for HRV analysis are described. Then, existing methods and the new procedure based on nonparametric bootstrap tests for median difference are provided in Section 3, together with the presentation of the available recordings in Physionet [11] used as starting benchmark for this study and the LTM-ECG databases for CHF and AF during 7 days. In Section 4, a set of experiments are conducted and results are presented on the suitability, together with some technical limitations and consistency properties, of these benchmarked multiscale algorithms. Finally, in Section 5, technical and clinical discussion is given and conclusions are drawn.

## 2. Fundamentals of Multiscale Methods for HRV Analysis

HRV measurements aim to give a numerical magnitude of the time fluctuations between sets of consecutive beats. The short-term recordings of HRV are usually measured about 3 to 5 min, and they have been traditionally associated with the dynamic control of the ANS on the heart rate and the cardiac properties. The long-term fluctuations of HRV have been described to have a wide physiological meaning in terms of the cardiovascular system self-regulation mechanism description. The ANS is divided into two branches, namely, the sympathetic and the vagal (parasympathetic) ones. Broadly speaking, the activation and excitation of the sympathetic branch has an accelerating effect on the cardiac cycle, whereas the vagal activation has a decelerating effect, but both subsystems are simultaneously and continuously working and compensating themselves, so that oscillations on a dynamic equilibrium are produced on the heart rate [9]. In addition, the ANS receives information through the so-called efferent pathways from a wide variety of systems and organs (heart, digestive system, kidney, respiratory system, and many others), and those influences are part of the genesis of ANS afferent pathways, in which heart rate is affected and is involved through different control mechanisms. Additional influences on the ANS such as humoral factors, night-day cycles, or environmental influences, are slower than the ANS, so that they only can influence the long-term HRV [30]. All these sources are contributors to the HRV modulation, which globally has its origin on a complex dynamic equilibrium arising from diverse mechanisms in the cardiovascular system that are taking place in the short-, the middle-, and the long-term scales.

A number of scientific, technical, and medical studies have focused on the HRV, and it well might be the most studied index in the SCD risk-stratification literature. Nonlinear methods [9] include several subfamilies, according to their calculation being based on Information Theory, on Chaos Theory, or on Fractal Theory. These methods have been paid special attention not only for their attractive theoretical foundations, but also because they seemed to have promising risk capabilities in small-sized studies with patients [7]. A usual situation in the literature of nonlinear HRV indices has been that some basic index has been first proposed, which has shown descriptive capabilities and some independence for SCD risk stratification, and then this index has been subsequently extended to a multiscale formulation, aiming to capture a richer variety of descriptions for the signal behavior. As described next, this is the case of MSE, MTI, and MFS methods for HRV analysis.

We denote the continuous-time ECG signal of a patient by S(t), and we register it during an observed time interval, denoted by $t\in (t_{i},t_{f})$. As a result of preprocessing steps devoted to signal filtering and R-wave detection, we can detect the R wave of each beat in S(t), and the time instants associated to each R-wave are denoted as $t_{n}^{R}$, with n=0,…,N, so that the detected set of R-waves can be expressed as a point process, given by

(1) $$RR\left(t\right)=\sum_{n=0}^{N}\delta \left(t-t_{n}^{R}\right)$$

where δ(t) denotes the continuous-time Dirac’s delta function. It is often useful to work with the so-called *normal beats*, which correspond to R-waves in beats that have been only originated in sinus rhythm conditions and where artifacts and ectopic beats are discarded. Here we assume that the R-waves correspond to cardiac beats but not to artifacts or wrong R-wave detections. The RR-tachogram [9], or just *tachogram*, can be denoted by x[n] and is defined as the discrete-time series given by the indexed time difference between consecutive R-wave times (excluding artifacts and non-physiological beats, as well as conventional quality-control beat filters), this is,

(2) $$x\left[n\right]=t_{n}^{R}-t_{n-1}^{R},\;n=1,\ldots ,N$$

The following algorithms and indices can be expressed and obtained in terms of the tachogram registered in patients.

### 2.1. MSE Analysis

The approximate entropy (ApEn) can be described as a nonlinear fluctuation measurement that aims to quantify the irregularity of a RR-interval time series [31]. An ApEn increase is usually interpreted as an indicator of irregularity increase in the underlying cardiovascular process. The Sample Entropy (SampEn) was subsequently introduced [32] to solve the limitations of the ApEn [33], since the latter compares each pattern in a time series with other patterns but also with itself, which leads to the overestimation of similarity existence in that time series, and hence to strong bias and to some inconsistent results. SampEn is the negative of the natural logarithm of the conditional probability that two similar patterns of *m* point segments, $x_{m}\left(j\right)$ and $x_{m}\left(i\right)$ of tachogram x[n], remain similar if we increase the number of points to m+1, within a tolerance *r* that is defined as a noise-rejection filter [32]. SampEn index reduces the statistical bias of ApEn index and it is a measurement rather independent of the data length, but its problem is that it it can be unstable when the counted events are scattered.

The new concept of multiscale analysis was proposed to overcome several limitations pointed out for ApEn and SampEn measurements which could be leading to clinical misinterpretation of HRV in some conditions. The MSE analysis was introduced by Costa et al. in [14], and it was also intended to provide a richer description of the cardiac dynamics in terms of a set of naturally related indices, rather than to use a single number. For a given discrete-time series, a new series is constructed in a scale τ, the terms of which are the average of the consecutive elements of the original series without overlapping. For a time series with τ=1, this corresponds to the original series, whereas for τ=2, the series is constructed with the average of the elements taken from two by two, and so on. We finally calculate the SampEn for each one of these new generated series. When the obtained values are represented versus the scale factor, the dependence of the measured entropy with the time scale can be scrutinized. The maximum scale to use depends on the number of samples in the time series.

Starting with tachogram signal x[n], we denote MSE as MSE(x[n],τ,r,m) to explicitly consider the dependence of its design parameters. We obtain the consecutive time series $y^{\tau }$, determined by scaling factor τ as follows:

- First, the original time series is divided into non-overlapping intervals with window size of τ samples. Then the signal mean is obtained for each of the sample windows.
- Each element of the series $y^{\tau }\left[j\right]$ is calculated according to the equation:
  (3) $$y^{\tau }\left[j\right]=\frac{1}{\tau }\sum_{n=n_{j}}^{j\tau }x\left[n\right],\;1\leq j\leq \frac{N}{\tau }$$
  where $n_{j}=\left(j-1\right)\tau +1$ for notation simplicity. For the first scale, the time series $y^{1}\left[j\right]$ is just the original time series. The length of each obtained time subseries is equal to $\frac{N}{\tau }$.
- The sample entropy index is calculated for each time series $y^{\tau }$, and it is represented as a scale-factor function SampEn(τ).

The MSE analysis has been applied to a variety of cardiac and cardiopathological situations, including the analysis of CHF [14], hypertensive and sino-aortic denervated conditions in experimental studies [24], and the ANS evolution before, during, and after percutaneous transluminal coronary angioplasty [34], as well as for prediction of ischemic stroke in patients with persistent AF [26], among other things. In all these cases, the length of the analyzed signals did not exceed 24 h using 20 as a maximum scale value, which involves x[n] tachograms with about or more than 20,000 samples each.

### 2.2. MTI Analysis

Time irreversibility has recently attracted attention in the cardiovascular-signal field. A signal is said to be time irreversible if its statistical properties change after its time reversal. The consistency loss of the statistical properties of a signal when the signal reading undergoes a change through time inversion is measured using the MTI, which represents an asymmetry index. This index is higher in healthy systems (with more complex dynamics) and it decreases in conditions like pathology or aging, as introduced in [15,16]. On the other hand, physiological time series generate complex fluctuations in multiple depending time scales, due to the existence of different hierarchical and interrelated regulatory systems.

The MTI analysis has been applied to measure nonlinear dynamics in heart-rate time series. For instance, MTI indices were computed in [35] for 20 healthy neonates to detect the presence of nonlinearity in their cardiac-rhythm control system, and temporal asymmetries were detected within their heart rate dynamics even shortly after birth.

### 2.3. MFS Analysis

Physiological signals have been shown to present fractal temporal structure under healthy normal conditions [12,36,37]. In particular, it was shown in [38] that time series generated by certain cardiovascular control systems in healthy conditions require a large number of exponents to adequately characterize their scaling properties, and that the nonlinear properties of this behavior are encoded in the Fourier phases. The same work used examples of CHF patients to contrast the previous finding with the loss of multifractality in this example of life-threatening condition. In this setting, RR-interval time series have been analyzed in terms of multiple scaling exponents. We next summarize the basic principles for estimating the MFS from RR signals that are usually followed in the literature. The interested reader can consult the original works on its application [19,38] and the details on the wavelet-transform modulus maxima method [39], which gives a principled calculation method for this purpose.

Whereas monofractal signals have the same scaling properties through time and they can be indexed by a single global exponent (such as Hurst exponent *H*) characterizing their fluctuations, other signals exhibit variations in their local Hurst exponent along time. When several subsets of a signal are characterized by the same local Hurst exponent $h_{o}$, and when each of these signals can be characterized by a fractal dimension measurement, we denote this estimated dimension as $D(h_{o})$. Accordingly, D(h) will have nonzero values on a set of discrete points in *h* for some class of signals. Local value of *h* is modernly estimated with Wavelet Theory [39], often using successive derivatives of the Gaussian function as the analyzing wavelet at different scales *a*, in order to remove polinomial trends with polinomial order up to the wavelet derivative order. In these conditions, the problem reduces to obtain the modulus of the maxima extrema of the time series wavelt transform at eath time instant.

We then estimate the partition function $Z_{q}\left(a\right)$, as the sumation of the *q*th powers of these local maxima as a function of scale, and for small scales, it is fulfilled that it has the form $Z_{q}\left(a\right)∼a^{\tau (q)}$, where τ(t) are exponents that can be estimated. In monofractal signals, a linear scaling exponent spectrum is obtained, given by τ(q)=qH−1. However, for multifractal signals we obtain a nonlinear expression, and it can be shown that τ(q)=qh(q)−D(h), where h(q)=dτ(q)/dq is not constant. Accordingly, the estimated fractal dimensions D(h) are obtained by the Legendre transform of τ(q), finally yielding

(4) D(h)=qh−τ(t)

Given that h=0.5 can be related to uncorrelated changing time series, this representation allows to determine to what extent a process conveys anticorrelated (h<0.5) or correlated (h>0.5) behaviour consistently manifested through different time periods.

The multifractal structure of HRV can reflect important properties of the heart-rate autonomic regulation. Multifractal analysis is an expansion of fractal analysis since it characterizes the time series variability with a collection of scaling exponents instead of a single one, which makes possible to investigate and quantify HRV in terms of its multiexponent properties. A right shift has been revealed in the multifractal spectrum peaks for healthy subjects during meditation [40], which points to a better health condition of persons with respect to multifractal nature. Accordingly, a healthy heart-rate regulation promotes a multifractal signal.

## 3. Statistical Methods and ECG Databases

### 3.1. Previously Proposed Indices and Bootstrap Resampled Median Difference

A set of metrics were computed in order to quantify population differences in terms of the information conveyed by small, medium, and large scales of MSE and MTI indices. Namely, the area under the MSE and MTI profiles between scales 1 and 5 (so-called Area 1–5), between scales 6 and 20 (Area 6–20), and between scales 21 and 100 (Area 21–100). The area under the complete profiles (so-called Area) for MSE, MTI, and MFS was also computed. These metrics have been previously used and validated in studies comparing results of multiscale indices for different populations [20,21]. The Wilcoxon-Rank Sum Test was subsequently used to evaluate the statistical difference between populations in terms of these metrics, also according to the previous works.

As an extension of the previous existing analysis, we contribute in this paper with a statistical procedure allowing to establish simple statistical comparisons, either between two different population groups or within the same group of patients, in terms of a given multiscale representation. The procedure can be summarized and described as follows. We generically denote the scale as ν (which includes both possibilities for τ in MSE and MTI, or *h* in MFS), and the multiscale index as J(ν) (where *J* stand for either MSE or MTI or MFS representations). Let us assume that we have available a set of signals from a given patient dataset *A*, and this set is denoted as

(5) $$S_{A}\equiv \left\{x_{i}\left[n\right],i=1,\cdots ,N_{A}\right\}$$

and that the multiscale parameter can be obtained by using a given operator Γ for each signal in the database, i.e.,

(6) $$J_{i}\left(\nu \right)=\Gamma \left(x_{i}\left[n\right],\theta \right)$$

where θ includes the set of preprocessing and processing parameters established for preprocessing and conditioning the signal under analysis.

Note that in this case J(ν) represents a random process, defined by its statistical distribution $f_{J(\nu )}\left(J(\nu )\right)$, which in general has an unspecified expression. We can define its median value and denote it as $J_{M}\left(\nu \right)$, which in practice can be estimated as the median of the multiscale representations obtained in a given population with a given set of parameters, and denoted as ${\bar{J}}_{M}\left(\nu |S_{A},\theta \right)$. Therefore, statistical differences can be calculated in two kinds of situations. First, when comparing the multiscale differences in two populations of patients, $S_{A}$ and $S_{B}$, we can build the statistic accounting for the difference between their corresponding poblational medians, give by

(7) $$\Delta J_{M}\left(\nu \right)={\bar{J}}_{M}\left(\nu |S_{A},\theta \right)-{\bar{J}}_{M}\left(\nu |S_{B},\theta \right)$$

In addition, we can have two different sets of preprocessing conditions, given by $\theta_{1}$ and $\theta_{2}$, and in this case the differences due to this change in those conditions can be scrutinized in terms of the difference of the median multiscale spectrum in a given population, as given by

(8) $$\Delta J_{M}\left(\nu \right)={\bar{J}}_{M}\left(\nu |S_{A},\theta_{1}\right)-{\bar{J}}_{M}\left(\nu |S_{A},\theta_{2}\right)$$

Hence, both representations are similar enough to provide a similar-to-handle view of the scales in which differences can be observed. The use of the median gives a robust estimator for cases where non-Gaussian distributions can be present, which was previously observed to be this case.

Since the *PDF* of the multiscale indices is often complex to estimate and to handle, we used nonparametric bootstrap resampling techniques, which provide us with an estimation of the empirical distribution of any statistical magnitude that can be built from computational media [29]. In our case, given a set of observed signals, we build a resample of this set by sampling with replacement each of the individuals in $S_{A}$ up to *B* times, so that we get the so-called the $b^{th}$ resample of the patient population, $S_{A}^{*}\left(b\right)$, where superscript ${}^{*}$ is the usual notation to point out all the bootstrap-estimated magnitudes. For this resample, we obtain an estimate of the statistical magnitude of interest, widely known as its bootstrap replication, and in our case it corresponds to weight vector $w_{\left(b\right)}^{*}$. By repeating the procedure *B* times, we get an estimate of the marginal distribution given by the empirical probability density function *(PDF)* of the bootstrap replications of each weight, this is, ${\bar{J}}_{M}\left(\nu |S_{A}^{*},\theta \right)$. The estimated distribution of the median difference statistic as a function of the scale can then be estimated from the replications of this statistic, which for the case of two different patient populations is obtained by non-paired resampling as follows:

(9) $$\Delta J_{M}^{*}\left(\nu ,b\right)={\bar{J}}_{M}\left(\nu |S_{A}^{*},\theta \right)-{\bar{J}}_{M}\left(\nu |S_{B}^{*},\theta \right)$$

whereas for changes in the preprocessing conditions, paired resampling can be addressed, yielding

(10) $$\Delta J_{M}^{*}\left(\nu ,b\right)={\bar{J}}_{M}\left(\nu |S_{A}^{*},\theta_{1}\right)-{\bar{J}}_{M}\left(\nu |S_{A}^{*},\theta_{2}\right)$$

The corresponding CI can be readily obtained just using sorted statistics, with significance level α yielding confidence level 1−α (typically 1−α=0.95). We expect the relevant differences to exhibit non-zero overlapping CI. In addition, the band confidence width should be consistent with the expected statistical power of the bootstrap test, hence allowing to study the consistency of the estimates when increasing the number of measured days in the Holter signals, and the estimated median average should allow us to scrutinize the presence of bias.

### 3.2. ECG Databases

We started by using multiscale methods to asses the variability of the RR-interval signals derived from 24-h Holter recordings from control subjects and from CHF patients. Both sets of recordings were downloaded from Physionet database [11]. The control group was obtained from 24-h Holter recordings in 72 healthy subjects (35 men and 37 women, from 20 to 76 years old). The original ECG recordings were sampled at 128 Hz. The CHF group was obtained from 24-h Holter recordings in 44 subjects (from 22 to 79 years old, including 19 men and 6 women, though gender information was not available for all the recordings). A subset of the original ECG recordings were sampled at 250 Hz (15 recordings), and the rest at 128 Hz. A number of studies have been conducted with these Databases [41,42,43,44] to determine the effect of exercise training on cardiac autonomic modulation in normal older adults using HRV, to establish normal values of RR variability for middle-aged persons, and to determine the effect of beta-blockers on parasympathetic nervous system activity.

We also used a specific LTM database, in which two sets of 7-day Holter recordings were also analyzed, one set from patients with CHF in sinus rhythm (73 recordings), and another set from patients with CHF with chronic AF (14 recordings). For short, we will denote them as CHF dataset and AF dataset, keeping in mind that both of them are CHF patients, but with different basal rhythms. The protocol to collect these recordings was carried out following the principles of Helsinki Declaration. It was approved by the Local Ethics Committee. Patients were recruited during scheduled outpatient visits to the CHF outpatient clinic in Virgen de la Arrixaca University Hospital (Murcia, Spain). From June 2007 to May 2011, patients with an established diagnosis of stable chronic CHF gave written informed consent to participate. All patients had LVEF ≤50% and they were clinically stable, without need for hospital admission or intravenous vasoactive agents within the past 3 months. Exclusion criteria included pacemaker-dependent patients, a serious comorbid condition with associated life expectancy <1 year, hospitalization for Myocardial Infarction (MI) and unstable Coronary Artery Disease (CAD) within the past 3 months, or any cardiac revascularization procedure within 30 days before enrolment. The 7-day continuous Holter recordings were obtained using a commercially available device (Lifecard CF^TM^, Del Mar Reynolds, Issaquah, Washington). These databases had been used [12,27,37,45] in previous studies: (a) To demonstrate that the circadian rhythms detected in 7-day recordings could not always be detected in 24-h periods; (b) to compare the diagnostic sensitivity of 1-day Holter monitoring versus 7-day Holter monitoring (7DH); (c) to detect atrial and ventricular arrhythmias in a population of stable patients with chronic HF and left ventricular dysfunction; (d) to characterize the relationship between heart rate and post-discharge outcomes in patients with hospitalization for HF with reduced ejection fraction (EF) in sinus rhythm; and (e) to characterize the infradian, circadian, and ultradian components for each patient, as well as circadian and ultradian fluctuations.

A standard Holter analysis software (ELA Medical^TM^, Sorin Group, Paris, France) was used to process the data. When needed, a trained cardiologist performed a visual check of the QRS complex classification and every arrhythmic event, therefore, manual corrections were made. Both data sets (Physionet and LTM) were preprocessed to exclude artifacts and ectopic beats, as follows. RR intervals lower than 200 ms and greater than 2000 ms were eliminated, as well as those which differed more than 20% from the previous RR interval [9]. The nonlinear indices were computed on the resulting time series.

## 4. Experiments and Results

In this section, we present a series of experiments in order to analyze the consistency and robustness of the indices for multiscale characterization with 1-day and 7-days Holter registers of healthy subjects, AF patients, and CHF patients. First, a robustness analysis of all these methods is made with datasets from Physionet databases (specifically, healthy subjects and CHF patients) in 1-day Holter recordings, and patients with AF and CHF are then analyzed in 7-day Holter recordings. We also scrutinized how the results can change when using 1-day Holter registers versus 7-day Holter registers and with increased scaling factor. A study on the robustness of the indices on 7-day recordings is checked in terms of 1-day segments, and a quantitative analysis is made for all of them in terms of the confidence bands for the populational medians.

### 4.1. Physionet Database and 1-Day Holter Recordings

**Figure 1:**
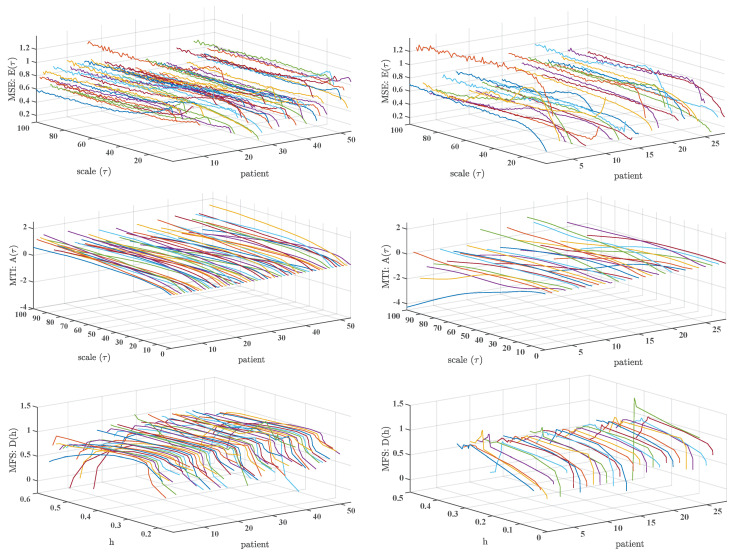
Results of multiscale analysis in Physionet Database: Multiscale Entropy (MSE) (**top**), Multiscale Time Irreversibility (MTI) (**middle**), and Multifractal Spectrum (MFS) (**down**) for the control database (**left**) and for the Congestive Heart Failure (CHF) patients (**right**).

We started our analysis by scrutinizing the effect of calculating multiscale indices in 1-day Holter recordings above 20 scales, which is the usual limit value in the literature. The multiscale indices were obtained in the 72 subjects in the control set of Physionet database. Figure 1 (left panels) shows these results, where each plot represents the entropy value (vertical axis) as a function of the multiscale parameter (τ or *h*), for every patient in the database and with no specific ordering. Typical patterns can be observed for each multiscale index. For instance, in MSE there is often a soft curve for low scales that usually turns to near constant at larger scales. We can observe also a rift effect specially in larger scales, which indicates that the method is being sensitive to noise. We can also observe a bias effect among subjects, as some of them appear to be above increased or decreased average levels compared with others. With respect to MTI, we can see that all the cases start near zero value for low scales, and there is a general trend to increase as a soft-changing curve above zero for all the cases. No rift effect is present in this multiscale index. Finally, MFS often shows an inverted-U shape, as documented in the literature, and there is a bow about h=0.5 in many cases, followed by a flat or slow decaying set of values. Note that some few cases seem to deviate from this populational behavior.

**Figure 2:**
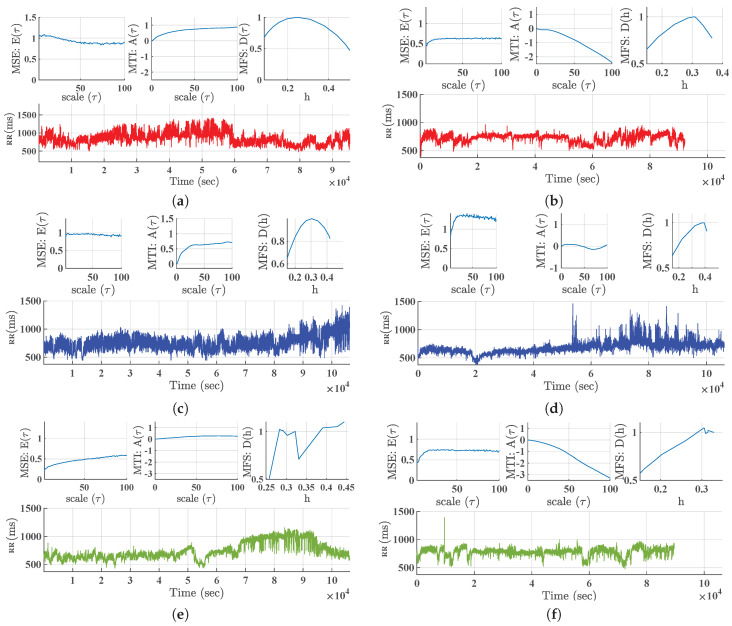
Results of multiscale analysis in example patients from Control Database (**left**) and from CHF Database (**right**) in Physionet: (**a**,**b**) Normal trend; (**c**,**d**) abnormal MSE and MTI profiles; (**e**,**f**) abnormal MFS profile.

A different behavior is present when we scrutinize the three multiscale indices for the CHF patients in 1-day Holter recordings, as seen in Figure 1 (right panels). This figure and its panels allow us to check the individual profiles of a given multiscale index in a population, so that individual profiles can be checked to be consistent with their population, and also non-concordant profiles can be clearly identified. Note that the axis are similar to the control panels, and that the vertical axis have been adjusted with the same range and scale. In MSE there is a diversity of shapes in low scales, though the trend to stabilize at larger scales is again observed, as well as the rift effect. In addition, there are more cases with increased average value, and in general there is a trend to exhibit lower average cases than controls. The MTI again starts from low values for low scales, but then it smoothly and often (not always) tends to go towards negative values. With respect to the MFS, it clearly decreases its width, the bow and the flat set of indices are mostly lost, and it often deviates from 1. Some very atypical cases are present, specially in the CHF set. Several patients are extremely different from the others in MSE and MTI, and several (not few) cases in MFS seem to present a breakdown and even values above 1. We thoroughly checked in all the patients that artifacts were correctly suppressed from the signals with ad-hoc designed software to represent jointly the RR-signals and the ECG signals on a similar time basis. Figure 2 presents six example cases (three from healthy subjects and three from CHF patients) in specific cases, namely, a normal-trend patient (typical MSE, MTI, and MFS) (Panels a, b), a patient with atypical MSE and MTI profiles (Panels c, d), and a patient with atypical MFS profile (Panels e, f).

### 4.2. Ltm Database and 7-Day Holter Recordings

**Figure 3:**
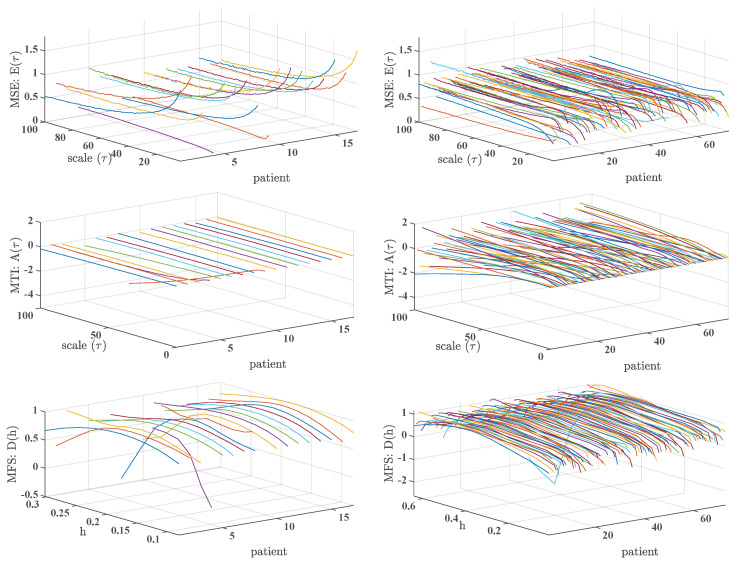
Results of multiscale analysis in long-term monitoring (LTM) database: MSE (**top**), MTI (**middle**), and MFS (**down**), for the atrial fibrillation (AF) database (**left**) and for the CHF database (**right**).

Figure 3 shows the MSE, MTI, and MFS results for AF and CHF 7-day Holter recordings databases. In the right panels, we can observe typical patterns that are similar to the CHF cases in 24-h recordings. In low scales, MSE exhibits a variety of shapes that tend to stabilize at larger scales. MTI indices show decreasing values from low to large scales reaching negative values in some cases. MFS shows a shape with an increased width and a lost of the bow. In the left panels, different trends are observed for AF patients. MSE shows an inverted shape compared to CHF patients, i.e., larger values for low scales and lower values for high scales, or a decreasing soft curve for low scales that usually turns into constant at larger scales. We can also observe a deviation of some of the subjects from the general trend. Regarding MTI, for low scales, values start near zero with a generally slight-decreasing trend curve. MFS shows an inverted-U shape with a bow about h=0.2 for all cases, followed by slowly decreasing values.

We designed a simple experiment accounting for windowing analysis of 7-day recordings in windows of 1-day duration. Our purpose here was to perform a robustness analysis of the 7-days Holter recordings when compared with 1-day recordings, in order to scrutinize the new knowledge provided by the scales up to 100 and to analyze the reproducibility of the multiscale indices throughout the 7 days.

**Figure 4:**
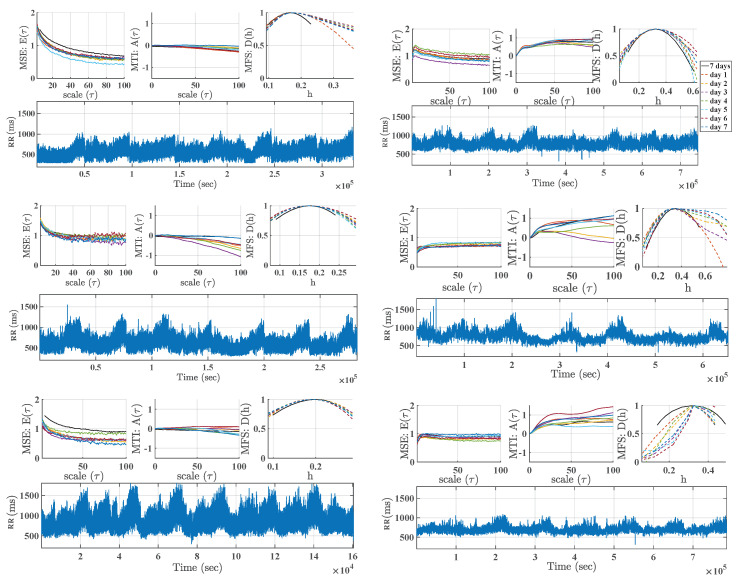
Examples of robustness analysis of 1-day versus 7-day Holter recordings in AF (**left**) and CHF (**right**) patients.

Figure 4 presents a comparative analysis between 1-day and 7-day recordings by means of 3 patients with AF and 3 patients with CHF. The heart-rate signal of each patient is presented as well as the MSE, MTI, and MFS profiles. Those profiles present similar trends for every 1-day segment and for the 7-day segment with variable variance depending on the particular case. However this variance is larger in CHF examples than in AF examples for MTI in large scales and mainly for MFS.

We can also check that the consideration of larger scales in the 7-day recordings has relevant effect on the estimation of the multiscale indices. For instance, the ripple in MSE is reduced in the 7-day estimations. In addition, the variance in the 1-day estimated indices for larger scales in MSE and MTI seem to stabilize to a profile which is not just the average of the consecutive days, which implies that nonlinear irregularity effects in these larger scales are present and likely they are better accounted by the 7-day calculations. It is interesting the effect that the use of 7-day recordings has on MFS, which is different from a simple day-averaging of the windowed spectra. In some cases, a wider set of 1-day spectra is condensed into a narrower spectrum with 7-day signals. In general, larger variance seems to be present at larger scales with 1-day signals, which is reduced by 7-day based spectrum (often yielding a spectral profile lower than the individual spectra in each day). Even in one case, apparently inconsistent daily spectra turn into a well-shaped spectrum when using the 7-day recording.

### 4.3. Statistical Analysis and Confidence Bands

Table 1 compares CHF patients and control subjects of Physionet database in terms of Area 1–5, Area 6–20, Area 21–100, and Area metrics for the multiscale indices. The Wilcoxon Rank-Sum Test reveals significant statistical differences for Area 1–5 in MSE and MTI, and for Area 6–20 in MTI, hence for small and medium scales. Table 2 shows the comparison for the two populations in the LTM database. In this case, statistical differences are present in MSE for small and medium scales, whereas in MTI these are also preset for large scales Area 21–100, and in MTI and MFS for the complete Area. The same statistical differences are observed when comparing the one day average results of LTM database (Table 3). Note that the interpretation of these differences should be taken as descriptive of the different cardiac conditions in terms of the different scales of the signal dynamics, and not as classifying characteristics for them with diagnostic purposes.

**Figure 5:**
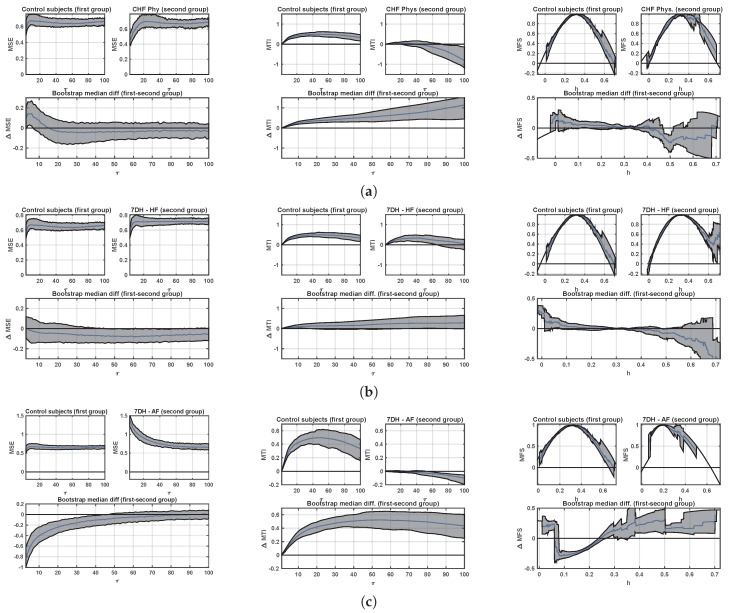
Confidence bands of multiscale analysis in control population vs different cardiopathy conditions: (**a**) Controls from Physionet (1D) vs HF patients from Physionet (1D); (**b**) controls from Physionet (1D) vs HF patients (7D) from LTM database; (**c**) controls from Physionet (1D) vs AF patients (7D) from LTM database. From left to right columns, the MSE, MTI, and MFS confidence bands and their differences are included.

Additional details can be scrutinized from the proposed method, aiming to extend the statistical behavior for the previous indices in different scales, populations and conditions, or scale span, in terms of confidence bands width and median differences. Figure 5 shows the confidence bands for the median multiscale indices when comparing the control set with the two considered cardiac conditions, namely, CHF and AF. The former is obtained from the Physionet CHF set and the later from the LTM-AF set. Each panel shows the median and confidence bands for the first group (up, left), for the second group (up, right), and for the median difference (down). Panel (a) shows that the MFS is mainly different between both groups for low scales in MSE, whereas significant differences are present in MTI differences in all the scales. For obtaining confidence bands in MFS, an interpolation was done to a regular grid sampling using chirp interpolation, and given the different scale spam for the obtained MFS in each patient, the confidence bands in each point of the scale grid was obtained conditional to the existence of the fractal spectrum in that scale. As it can be seen in the right panel, the control set has a scale span between 0 and 1, with some exceptional case extending out of it, but the confidence band gets wider after 0.6, whereas the scale span is mainly narrower in CHF patients, though sometimes it reaches a similar set of values for different patients, as it can be observed from the wider confidence bands in the left and specially in the right of the graph. The median difference indicates a clear descending trend in the median value (blue line) from left to right, which is only non-overlapping zero at about scale 0.5. Panel (b) in Figure 5 shows the result of a similar comparison in this case between the control subjects from Physionet (1D) and the CHF set from the LTM database (7D). The relevant differences with respect to the previous comparison can be summarized as follows. For MSE, the differences in the lowest scales are less present, and there is a trend to the band to be consistently below zero, though it remains non-significantly yet borderline. For MTI, the differences between both populations show a similar trend than in the previous comparison, though the confidence bands in this case are borderline with respect to their zero overlapping. For MFS, the confidence bands in the CHF population from 7D reaches a wider interval of scale values remaining narrow, and the confidence bands for the median difference exhibit a similar trend with smoother band limits than the previous comparison. Panel (c) in Figure 5 shows the comparison between the control subjects from Physionet (1D) and the AF set from the LTM database (7D). For MSE, significant differences are present in low scales (below 60), whereas for MTI there are significant differences in all the scales. In addition, significant differences are present in MFS in almost all the scales, whereas in this case the median trend has the opposite slope than in the preceding comparisons with CHF. Whereas several of these results have been previously documented in the literature, our results are consistent with those precedents and they can be observed at a glance from the representation. It is evident that these indices are measuring different aspects of the complexity and/or nonlinear nature of the cardiac dynamics, and that they probably should be used complementarily.

**Figure 6:**
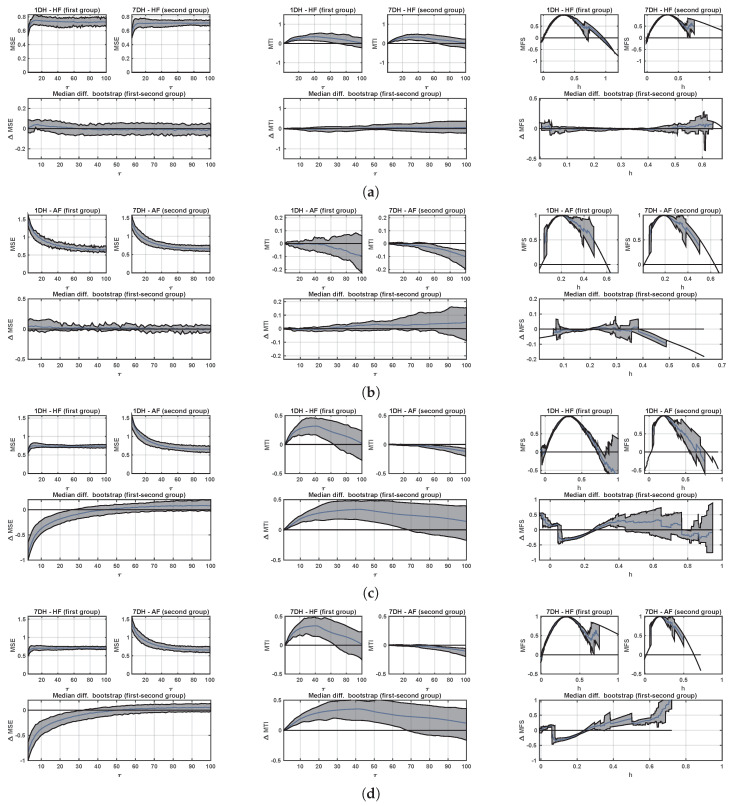
Confidence bands of multiscale analysis in LTM database: (**a**) Paired 1D vs 7D in HF patients; (**b**) paired 1D AF vs 7D in AF patients; (**c**) non-paired 1D HF vs 1D AF patients; (**d**) non-paired 7D HF vs 7D AF patients. From left to right columns, the MSE, MTI, and MFS confidence bands and their differences are included.

Figure 6 shows the confidence bands of multiscale analyses with the aim of establishing the statistical properties that can be expected due to the use of LTM recordings. In this setting, and as it could be expected, the confidence-band widths are consistently narrower when scrutinizing paired datasets, i.e., those comparisons in which the indices estimated from 7-day recordings are compared to the indices estimated in one day (the third in each set for all of them in the 7D register) of 1D recordings for the same patients. Panel (a) shows the scale-paired confidence bands for the median differences when analyzing the CHF patient set, showing a trend to raise in the very low scales, which could mean that the significance of these scales depends on the number of days considered, when working with CHF patients. The estimated MTI remains very reproducible when obtaining it from 1D or from 7D in each patient. Note the different behavior of MSE and MTI in terms of the confidence band width in terms of increasing scales (mostly constant in MSE and increasing error standard with scale in MTI). With respect to MFS, whereas the median value difference is not significant, the most critical scale regions are again the boundaries of the population spectrum, in terms of confidence band widths. Similar conclusions can be obtained for the AF patients when compared in terms of MSE and MTI. The scale-border effects is even more critical in this case, as AF wide of this spectrum is narrower in the patients compared with control subjects and with CHF condition patients. Panels (c) and (d) show the result of comparing different conditions (CHF and AF) when using 1D or 7D in these two different sets of patients. Note that in this case the confidence bands of the differences are not very different for the cases of MSE and MTI, whereas both the confidence band widths and the scale span are clearly narrower when they are compared in terms of 7D recordings.

## 5. Discussion and Conclusions

In this work, we have addressed the calculation of three representative multiscale indices, namely, MSE, MTI, and MFS, on 1-day and 7-day Holter recordings. From our results, we can conclude that, when present, the trends are consistent between the additional scales provided by 1-day recordings and by the 7-day recordings, but the second ones can give a statistically better view of this kind of representations, specially in the MFS representations.

*Contributions.* Several preceding results can be found in which descriptions of 1-day estimated indices are shown, however, and to the best knowledge of the authors, few works address from this perspective the 7-days case with scales up to 100. Accordingly, and given the advances in the monitoring and well-being technology in our days, this represents a good moment to determine the possible advantages and issues of using these methods in larger observation scales. Non-linear methods used in this work (MSE, MTI, and MFS) have previously been proposed and widely used in the literature. The contributions of our work are twofold. On the one hand, we analyzed the dynamics, the consistency, and especially the robustness of these indices when applied to the new long term scenarios (7 days) and to point out that algorithms may need to be improved when using them in this area. On the other hand, to find out if further information is conveyed in the temporal scales that have not been analyzed before in shorter recordings.

*Summary and Discussion of Results.* Results showed significant differences between CHF and AF populations not only for short-term scales but also for long-term and very long-term scales in some indices, MSE (MTI) being higher (lower) for AF. These results are consistent with previous studies [14,15,16]. Here we confirmed the same trends, but we also obtained several differences in very long-term scales that had not been analyzed before. MSE was not further significantly different for very long-term scales. This may be attributed to the fact that statistical characteristics of AF signals resemble those from noise, and being MSE a entropy-based measure, it is higher for AF than for CHF for short-term and long-term scales, but not for very long-term scales, where the surrogate signals present attenuated these erratic characteristics. On the other hand, MTI shows that time irreversiblity is for all time scales lower in AF patients, i.e., for those presenting a more severe pathology.

We detected that 1-day estimations of the multiscale indices can show a distorted profile. The different estimations observed in the different time windows could be due to several reasons. In addition, this effect could have statistical roots, in terms of consistency and variance of the estimation with shorter time series. On the other hand, it also could be due to the fact that some frames had some properties in their dynamics which made the multiscale algorithm fail or disrupt. The differences in daily activities should not be a limitation, as far as the multiscale algorithm measurements are the correlations present in the signal at different time scales. In the conventionally used surrogate-signal test, which is built with lower-pass versions of the original signal, the more we increased the scales, the more the short-term relationships were eliminated to manifest the long-term. Thus, the short-term differences would be hidden for high scales. We thoroughly checked that the underlying signals did not have artifacts or aberrations with a specifically customized software to support an observer to watch at the heart-rate signals and at the original ECG signals simultaneously. Accordingly, one of our conclusions is that these multiscale algorithms, while likely informative, should trust not only on the increase of the length of the signals for their consistency, but also on improving their algorithmic robustness. While stationarity can not often be assumed for HRV signals, MSE was originally proposed and applied in 24-h Holter recordings, and later many other works have used MSE to study different dynamics in 24-h. The analysis of the underlying dynamics during the day or night can be complex with these methods. Nevertheless, we observed that the disruption effect in one day does not have any significant impact in the 7-day analysis, which promotes their use in favor of improved statistical consistency.

*Usefulness of Median-difference Tests.* An extension of previously proposed statistical comparisons for scales has been proposed here in terms of nonparametric bootstrap resampling, which allows us to establish poblational comparisons in terms of the median difference of the multiscale representations, either for different health conditions, or for different acquisition conditions. Our results with this method were consistent with the results identified in the literature and showed some non-observed differences, specially the ones related with AF patients, and with different statistical consistency behavior and retrieved information about the patient given by these three multiscale indices, which suggests their use jointly in these and other populations.

*Related Relevant LTM Applications* With the technological availability of cardiac monitoring systems for extended time periods, LTM is expected to bring interest to new applications in health, wellness, and research. For instance, it has been recently pointed out [30] that energetic environmental phenomena can affect psychophysical processes on people in different ways depending on their sensitivity, health status, and ability of the ANS to self-regulate. In that study, the HRV was recorded for 72 consecutive hours per week over a five-month period in 16 participants, in order to examine ANS responses during normal background environmental periods. Interestingly, HRV measurements were negatively correlated with the solar wind speed, and the low-frequency and high-frequency power were negatively correlated with the magnetic field. This study confirmed that the daily activity of the ANS responds to changes in geomagnetic and solar activity during periods of undisturbed normal activity, it starts at different times after changes in various environmental factors, and it persist for variable time periods. As far as the activity of the ANS reflected by HRV measurements is affected by solar and geomagnetic influences, the analysis of HRV should take into account these effects when possible. This study is focused on the ANS modulation, and hence spectral measurements were mostly used, nevertheless, this kind of data could be analyzed with multiscale indices to provide a wider view on the long-term behaviour of HRV.

*On the Clinical Usefulness of Multiscale Indices.* As described with detail in [7], many indices have been proposed in the last years to develop risk stratification from different kinds of analysis of electric cardiac signals. However, these techniques from the academic research world rarely are used in the clinical practice. In that reference, our team analyzes the possible reasons by decoupling the sometimes limited accuracy and the lack of consensus on the robustness with the appropriate signal processing implementations. In this line of search, the present work aims to first establish the need for robustness in the methods that are widely used nowadays, as a requirement before enrolling in risk stratification studies, which require high-cost and high effort to yield clinically useful use to these techniques. Our future research would consider first to perform the same multiscale analysis (MSE, MTI, and MFS) in LTM healthy subjects, in order to study the dynamic behavior in normal conditions when increasing the number of scales. As indicated, the development of more robust multiscale indices is a desirable target in order to continue to progress in this informative characterization of the cardiac dynamics. On the other hand, AF and CHF are different heart diseases with different dynamics, though in this study we only could analyze AF patients as a subset of CHF patients. CHF is a syndrome of the deterioration in short-term and long-term regulatory mechanisms, while AF, and especially the persistent one, is an intrinsic short-term mechanism. Whereas long-term mechanisms can be present in AF, it seems that LTM recordings should be better used to analyze their presence or deterioration.

Despite the need for more robust algorithms in long-term nonlinear indices, we still consider that it worth the effort to use these indices, as vast literature supports their informativeness in other scenarios in addition to the very long-term monitoring. In general terms, the underlying hypothesis in preceding studies is that heart rate oscillations respond to phenomena with very different characteristic times, ranging from seconds to months. The former are well evaluated in a 24-h recording, but the low frequency (such as circadian cycles, many hormonal cycles, or secondary to changes in activity during the week) would only be represented in long-term monitoring. The response to these stimuli has been moderately studied, but it could provide us with interesting information on some clinical aspects, such as the prediction of decompensation in heart failure, the evolution of cardiovascular remodelling after acute injury (e.g., in the weeks following acute myocardial infarction), the evolution of sports training, or the susceptibility to the development of malignant arrhythmias (related with sudden cardiac death). All these aspects involve hormonal processes, inflammatory processes, adaptations of different organs or systems, and they are characterized by the interaction of mechanisms with slow response times, so that they develop in days or weeks, rather than in minutes or hours. Therefore, methods of analyzing the very long-term behavior of heart rate responses could be of interest, especially in noisy rhythms such as AF, in which almost all the usual HRV parameters are difficult to interpret.

We still do not know if these methods will be widely used in future clinical practice because we are still in the phase of describing how these indices behave, their variability and their comparison with the results in 24 h, among other thing. In any case, all these efforts should be supported by robust algorithms for multiscale nonlinear indices, an idea that had not been previously paid much attention in the nonlinear HRV literature.

## Figures and Tables

**Table 1:** Physionet database, congestive heart failure (CHF) versus Control. Area 1–5, Area 6–20, Area 21–100 and Area metrics expressed as mean ± standard deviation for the multiscale indices. Significant statistical differences given by the Wilcoxon Rank-Sum Test are indicated.

Scale Test	CHF	Control	*p*-Value
MSE (Area 1–5)	1.30 ± 0.50	1.58 ± 0.57	<0.05
MSE (Area 6–20)	9.47 ± 3.52	9.66 ± 2.75	0.53
MSE (Area 21–100)	56.45 ± 18.23	52.37 ± 11.88	0.38
MSE (Area)	68.51 ± 22.30	65.02 ± 15.27	0.69
MTI (Area 1–5)	0.09 ± 0.09	0.20 ± 0.14	<0.05
MTI (Area 6–20)	1.95 ± 1.79	4.41 ± 2.37	<0.05
MTI (Area 21–100)	50.46 ± 35.72	43.54 ± 27.76	0.35
MTI (Area)	52.77 ± 36.54	48.71 ± 29.77	0.68
MFS (Area)	0.32 ± 0.41	0.27 ± 0.09	0.62

**Table 2:** Long-term monitoring (LTM) database, atrial fibrillation (AF)-7-day Holter monitoring (7DH) versus heart failure (HF)-7DH. Area 1–5, Area 6–20, Area 21–100 and Area metrics expressed as mean ± standard deviation for the multiscale indices. Significant statistical differences given by the Wilcoxon Rank-Sum Test are indicated.

Scale Test	AF-7DH	HF-7DH	*p*-Value
MSE (Area 1–5)	3.01 ± 1.16	1.55 ± 0.47	<0.05
MSE (Area 6–20)	12.89 ± 4.89	10.22 ± 2.63	<0.05
MSE (Area 21–100)	52.34 ± 18.03	56.63 ± 12.44	0.79
MSE (Area)	70.16 ± 24.51	69.80 ± 15.53	0.27
MTI (Area 1–5)	0.04 ± 0.04	0.16 ± 0.13	<0.05
MTI (Area 6–20)	0.72 ± 1.61	3.13 ± 2.17	<0.05
MTI (Area 21–100)	19.74 ± 50.62	42.78 ± 25.18	<0.05
MTI (Area)	20.60 ± 52.31	46.47 ± 26.29	<0.05
MFS (Area)	0.25 ± 0.12	0.48 ± 0.91	<0.05

**Table 3:** LTM database, AF-1-day Holter monitoring (1DH) versus HF-1DH. Area 1–5, Area 6–20, Area 21–100 and Area metrics expressed as mean ± standard deviation for the multiscale indices. Significant statistical differences given by the Wilcoxon Rank-Sum Test are indicated.

Scale Test	AF-1DH	HF-1DH	*p*-Value
MSE (Area 1–5)	3.06 ± 1.18	1.63 ± 0.58	<0.05
MSE (Area 6–20)	12.96 ± 4.74	10.58 ± 3.24	<0.05
MSE (Area 21–100)	50.58 ± 17.37	56.68 ± 14.16	0.28
MSE (Area)	68.54 ± 23.48	70.35 ± 18.11	0.82
MTI (Area 1–5)	0.04 ± 0.06	0.18 ± 0.17	<0.05
MTI (Area 6–20)	1.12 ± 2.69	3.41 ± 2.47	<0.05
MTI (Area 21–100)	31.68 ± 87.92	45.79 ± 30.89	<0.05
MTI (Area)	33.00 ± 90.97	49.82 ± 32.48	<0.05
MFS (Area)	0.26 ± 0.11	0.45 ± 0.51	<0.05
